# Dynamic immobilization of bacterial cells on biofilm in a polyester nonwoven chemostat

**DOI:** 10.1186/s40643-024-00732-0

**Published:** 2024-01-24

**Authors:** Chao-Lei Zhang, Chao Wang, Yue-Sheng Dong, Ya-Qin Sun, Zhi-Long Xiu

**Affiliations:** 1 School of Life Science and Biotechnology, Dalian University of Technology, Dalian, 116024 People’s Republic of China; 2 Public Security Management Department, Liaoning Police College, Yingping Road 260, Dalian, 116024 People’s Republic of China; 3 School of Environmental Science and Engineering, Sun Yat-Sen University, Guangzhou, 510006 People’s Republic of China

**Keywords:** Cell immobilization, Biofilm, Adsorption/desorption, Extracellular polymeric substances

## Abstract

Cell immobilization plays an important role in biocatalysis for high-value products. It is necessary to maintain the viability of immobilized cells for bioconversion using viable cells as biocatalysts. In this study, a novel polyester nonwoven chemostat was designed for cell immobilization to investigate biofilm formation and the dynamic balance between adsorption and desorption of cells on polyester nonwoven. The polyester nonwoven was suitable for cell immobilization, and the cell numbers on the polyester nonwoven can reach 6.5 ± 0.38 log CFU/mL. After adding the polyester nonwoven to the chemostat, the fluctuation phenomenon of free bacterial cells occurred. The reason for this phenomenon was the balance between adsorption and desorption of bacterial cells on the polyester nonwoven. Bacterial cells could adhere to the surface of polyester nonwoven via secreting extracellular polymeric substances (EPS) to form biofilms. As the maturation of biofilms, some dead cells inside the biofilms can cause the detachment of biofilms. This process of continuous adsorption and desorption of cells can ensure that the polyester nonwoven chemostat has lasting biological activity.

**Graphical Abstract:**

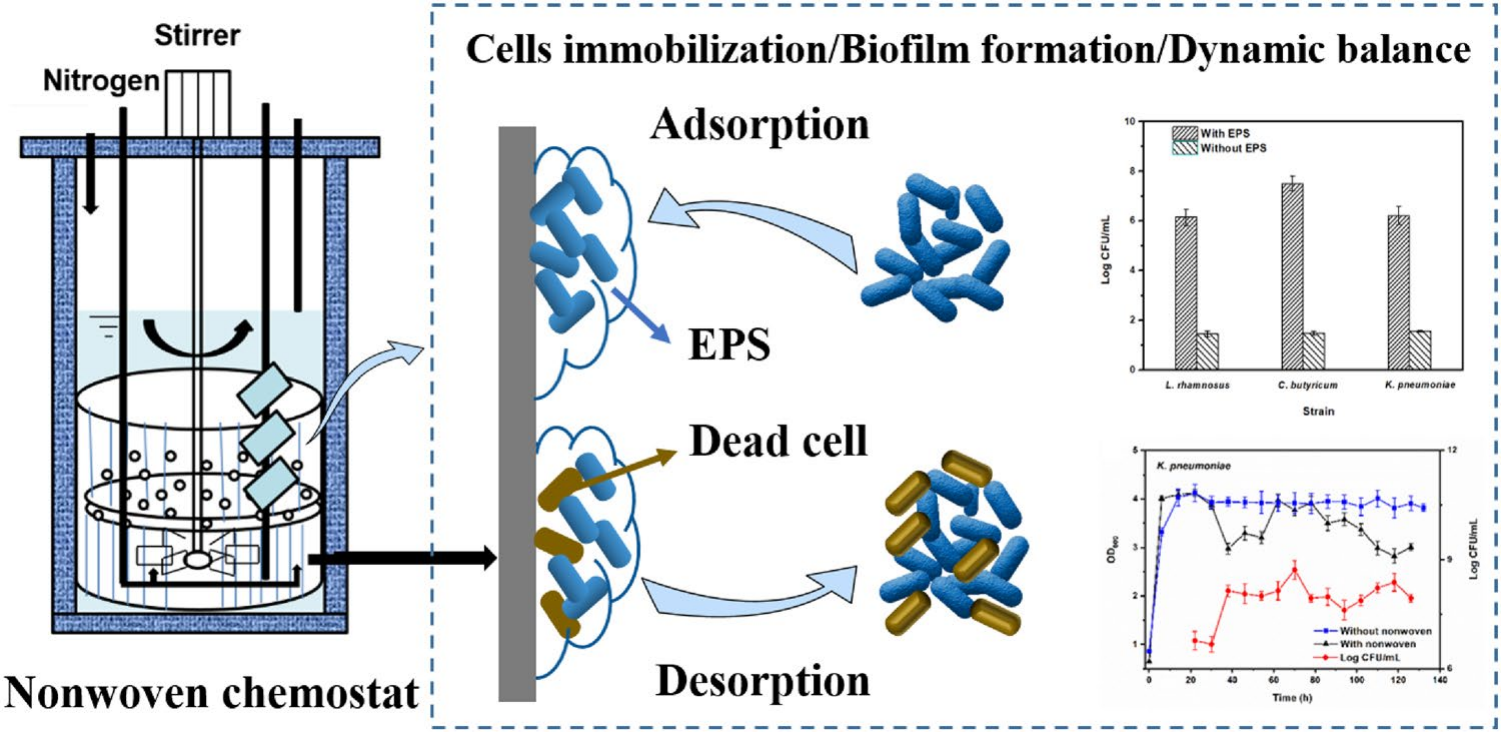

## Introduction

Microorganisms are crucial for many scientific and industrial applications in the biofuel production pharmaceutical, and food industries (Zhu 2007; Karagoz et al. 2009). Microbial fermentation, the main step in target product generation, is the conversion of substrates (e.g., sugars, glycerin, food waste, and agricultural wastes) into target products (e.g., bioethanol, 1,3-propanediol, lactic acid, and *n*-caproate) and carbon dioxide with the help of fermenting microorganisms. Microbial cell suspension systems are the most common way for microbial fermentation, but suspended cultivation systems permit relatively low cell densities, low feed rates in continuously feeding processes, and hardly any separation of cells from the bulk liquid (Zhang et al. 2019; Karagoz et al. 2019). Therefore, the immobilization technique is developed to overcome the disadvantages of the suspended cells.

The immobilization technique is the attachment or inclusion of the microbial cells or enzyme in a distinct solid support (carriers) that enables the exchange of substrates and products while separating the cells or enzyme at the same time from the bulk phase (Taheri-Kafrani et al. 2021). Cells or enzyme immobilization will require a lower reaction volume, ease the separation of cells or enzyme, form products with higher purity, and facilitate higher feeding rates without washing out the free cells or enzyme (Tran et al. 2015; Behera and Ray 2015; Ma et al. 2017; Aghaei et al. 2022). Furthermore, the immobilizing microorganisms or enzyme can withstand the harsh environment of media, such as variable temperature, pH, and shear stress (Cassidy et al. 1996; Park and Chang 2000; Taheri-Kafrani et al. 2021). At present, physical or chemical approaches, such as trapping and adsorption, or cross-linking and covalent attachment, are the two most frequent ways to immobilize cells or enzyme (Mortazavi and Aghaei 2020). The most popular tactic is physical approaches since it is easy to implement and does not require functionalization support (Aghaei et al. 2022). For example, entrapment is one of the most widely used immobilization techniques for cells, and calcium alginate is the common embedded material. The immobilization of *Saccharomyces cerevisiae* has been investigated by entrapment in calcium alginate beads for optimization of bioethanol production (Mishra et al. 2016). After immobilizing *Bacillus subtilis* in alginate microparticles, the production of lipase was almost three times higher than free cell cultivation (Oliveira et al. 2019).

Compared to entrapment, another popular immobilization technique is the adsorption of cells or enzymes on the carriers' surface. The carrier’s properties are critical for a successful immobilization process. The carrier must be chemically, thermally, and mechanically stable, as well as insoluble in the immobilization solution (Hartmann and Kostrov 2013). In addition, the support needs to be reasonably priced, safe for the environment, and prevent the cells or enzymes from becoming denaturated or deactivated (Gao 2006). Recently, the immobilization materials for cells and enzymes involve rice husk, carrageenan, natural sponge, porous cellulose, tosylated cloisite, epoxy-activated cloisite (ECL), cloisite 30B, and polyester nonwoven (Liu et al. 2023; Nezhad and Aghaei 2021; Ogbonna et al. 2001; Sakurai et al. 2000; Aghaei et al. 2021, 2022; Zhang et al. 2021a). For enzyme immobilization, the specific activity of α-amylase adsorbed on cloisite 30B was 2.39 ± 0.03 U/mg proteins at the optimum pH 8 (Aghaei et al. 2022). Moreover, the hydrolysis of olive oil, the synthesis of isoamyl acetate, and the generation of biodiesel all employed lipase immobilized on the ECL (LECL). Under ideal circumstances, the greatest production of ester and biodiesel was 95.4% and 1.85 ± 0.05 U/mg for LECL’s hydrolytic activity (Aghaei et al. 2021). Unlike the immobilization of enzymes, the immobilization of cells typically leads to the formation of biofilms on carriers. When *Clostridium kluyveri* was immobilized on the wheat straw carrier, the biofilm formed showed high activity for caproate production and provided tolerance to ammonia inhibition (Zhang et al. 2019). The polyester nonwovens were frequently employed for cell immobilization because of easy formation of biofilms. When the cells were immobilized in a polyester fibrous matrix, a high viable cell density of 3 × 10^8 ^cells/cm^3^ packed bed was achieved, resulting in a high volumetric monoclonal antibody productivity of 1 g/(L·day) under continuous feed circumstances (Yang et al. 2004). The polyester nonwoven chemostat can also be applied to investigate the effect of probiotics on pathogens (Zhang et al. 2021b). However, the previous studies solely looked at the generation of metabolites by immobilization cells and overlooked the dynamic balance between the adsorption and desorption of immobilized cells.

In this study, a polyester nonwoven was incorporated into a chemostat to immobilize the microbial cells. The main objectives of this study were to (i) assess the feasibility of cell immobilization in the polyester nonwoven chemostat; (ii) investigate the dynamic balance between adsorption and desorption of cells on polyester nonwoven; (iii) evaluate the role of extracellular polymeric substances (EPS) in cell immobilization.

## Materials and methods

### Strains and media

The strains *Clostridium butyricum* (CGMCC 0313-1) producing butyric acid and 1,3-propanediol and *Klebsiella pneumoniae* (CGMCC 10438) producing 1,3-propanediol and 2,3-butanediol were obtained from the China General Microbiological Culture Collection Center (Beijing, China). *Lactobacillus rhamnosus* (CICC 22825) producing lactic acid was obtained from the China Center of Industrial Culture Collection (Beijing, China). All strains were maintained in stock culture, kept in 20% glycerol at −75 ℃. The cultural medium was based on previous studies (Pan et al. 2008). Medium was prepared as follows (g/L): 20 g of glucose, 5 g of sodium acetate, 5 g of yeast extract, 10 g of peptone, 0.2 g of MgSO_4_, 1 g of ammonium citrate, and 0.05 g of MnSO_4_ per liter. The chemicals used in this study were all purchased from Shanghai Macklin Biochemical Technology Co., Ltd.

### Establishment of a polyester nonwoven chemostat

**Fig. 1:**
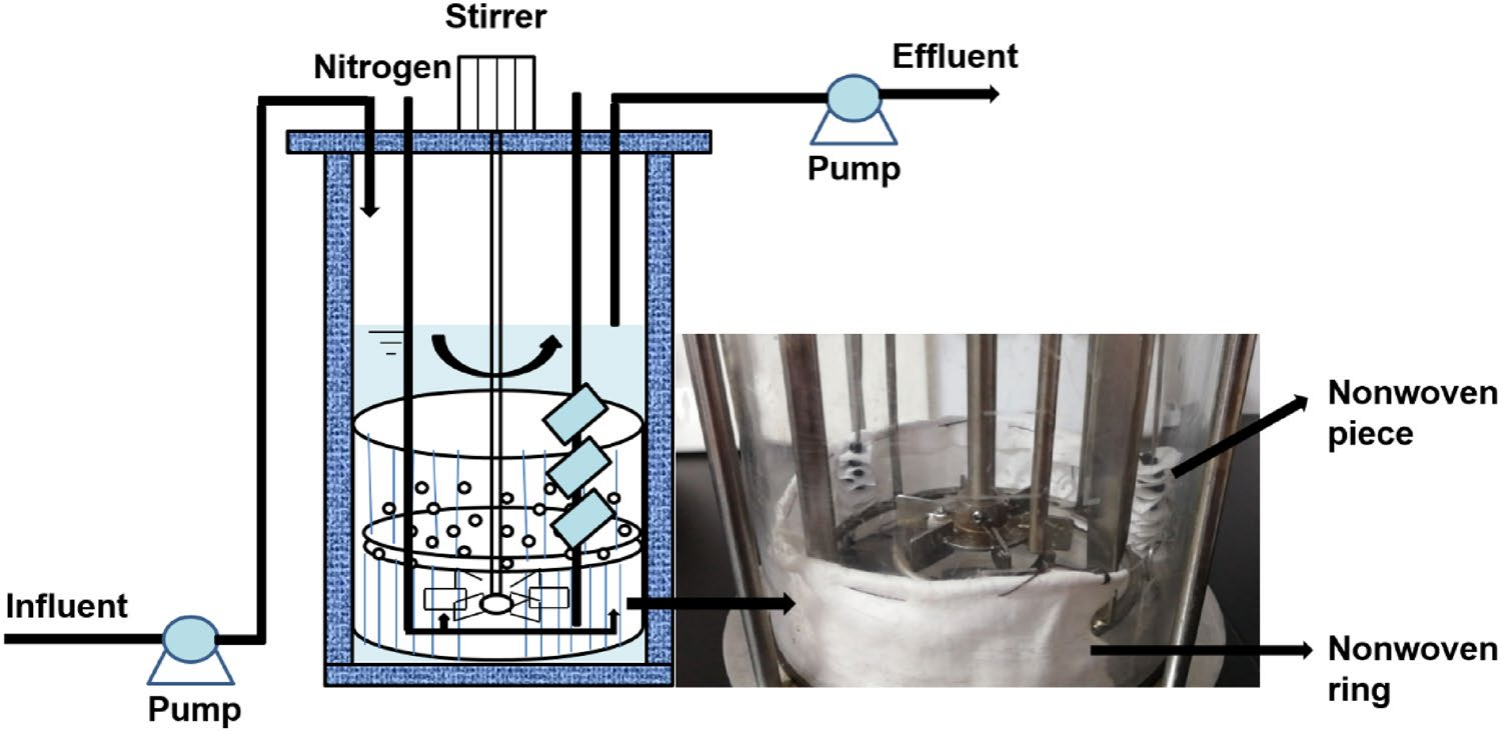
Schematic diagram of the polyester nonwoven chemostat

A polyester nonwoven chemostat was established by employing the intake and output pumps to maintain a constant liquid level in the fermenter. The fermenter was purchased from Shanghai Baoxing Bio-Engineering Equipment Co., Ltd. (China). The chemostat held 1.0 L worth of culture in total. The dilution rate was fixed at about 0.045 h^−1^. By automatically adding sodium hydroxide, the fermenter’s pH was kept at 6.0 and the temperature was maintained at 37 ℃ (Likotrafiti et al. 2014). The agitation rate was 150 rpm. To keep the chemostat under anaerobic conditions, 100% nitrogen was flushed through it. Compared with other materials, polyester nonwoven is more inclined to bind to cells due to its pore spaces for maintaining moisture (Colclasure et al. 2015). The nonwoven was purchased from Jiangxi Gemei Medical Equipment Co., Ltd. (China). The raw material for nonwoven is composed of polyester, polypropylene, acrylic fiber, and viscose. A steel ring seaming with polyester nonwoven (35 × 10 × 0.2 cm) was incorporated into the chemostat (Fig. 1). At the same time, to analyze the dynamic process between biofilm and suspended cells, the polyester nonwoven piece was simply inset into the chemostat, as shown in Fig. 1. The area of the polyester nonwoven piece was 4 × 4 cm to take them from the chemostat conveniently. When taking the sample of the polyester nonwoven piece, the room where the chemostat was located was sterilized by ultraviolet for 30 min, and then the flame ring was used to ensure the sterility around the sampling port of the chemostat. The sterilized tweezer was used to remove the polyester nonwoven piece from the stainless steel. The fermentation experiment for each strain was repeated three times.

### Analytical methods

The optical density (OD) at 600 nm was measured using a Shimadze UV–VIS-NIR Spectrophotometer (UV-3600) with a 2 mL fermentation sample that was obtained from the fermenter every 8 h. Every 8 h, a polyester nonwoven piece (4 × 4 cm) was taken from the fermenter and washed with 2 mL of sterile water before being used for serial dilutions and plate counts to determine the number of cells.

### Preparation of PMA treatment samples

Treatment of bacterial samples with propidium monoazide (PMA) has been successfully used to differentiate viable and non-viable cells in conjunction with qPCR (Nocker et al. 2007; Vesper et al. 2008). PMA can enter compromised membranes and intercalate the DNA, or it can intercalate into extracellular DNA (Tavernier and Coenye 2015). In this study, a PMA-qPCR test was performed to analyze the viable and non-viable cells on the polyester nonwoven. PMA (Biotium, USA) was dissolved in 20% dimethyl sulfoxide (DMSO) to a concentration of 20 mM, and aliquots were transferred to 1.5 mL shaded micro-centrifuge tubes for storage at − 20 ℃. All preparation processes for PMA stock solutions were performed under minimal light. The cells were rinsed from the polyester nonwoven into a 2-mL centrifuge tube with sterile water, and the cell suspensions were centrifuged at 12,000 rpm for 10 min. Cell pellets were used for analysis by the PMA-qPCR method. The cell pellets were first washed twice with PBS and resuspended in 462 μL of sterile water. Then, a total volume of 38 μL of PMA stock solution was added to 462 μL of sample in the centrifuge tube (final PMA concentration of 30 μM) and held for 10 min in the dark at room temperature with periodic mixing. The sample tubes were exposed horizontally on ice for 10 min at a distance of 20 cm from a 500-W tungsten halogen lamp. After light exposure, cells were centrifuged at 12,000 rpm for 5 min for the DNA extraction described below. The proportion of death cells was calculated by the following equation:

$$\mathrm{Proportion \; of \; death \;cells}=\frac{{Copies \;number}_{qPCR}-{Copies\; number}_{PMA-qPCR}}{{Copies \;number}_{qPCR}}.$$

### DNA extraction and qPCR analysis

Genomic DNA from the PMA-treated samples was extracted with the TaKaRa MiniBEST Bacterial Genomic DNA Extraction Kit Ver.3.0 (Takara Bio Inc., Dalian, China), according to the manufacturer’s instructions. The purity and quantity of DNA were determined with a Nanodrop1000 spectrophotometer (Thermo Scientific, Wilmington, DE, USA). The PCR assay was conducted on a 7500 Fast Real-time PCR System (Applied Biosystem, Foster City, CA, USA). PCR amplification was carried out in a total volume of 20 μL containing 2 μL DNA templates and an 18 μL PCR mixture. The PCR mixture included 10 μL SYBR Premix Ex Taq^™^ (Takara Bio Inc., Dalian, China), 0.4 μL ROX Reference Dye II, 0.8 μL (0.4 μM) forward primer and reverse primer, and 6 μL ddH_2_O. The amplification conditions comprised the following steps: at 95 ℃ for 30 s, 40 cycles at 95 ℃ for 5 s, and 60 ℃ for 30 s. The primers used for qPCR amplification are shown in Table 1 (Sun et al. 2010; Laurinavichene et al. 2010; Larsen et al. 2013). Using the Student’s *t*-test, statistically significant differences between groups were assessed.

**Table 1:** Quantitative PCR primers for quantification of *C. butyricum*, *K. pneumoniae* and *L. rhamnosus*

Primer	Target	Sequence of probe (5ʹ–3ʹ)	Refs
Reverse	*C. butyricum*	CCGGGCAGTCTCGCTAGAGTG	Laurinavichene et al. 2010
Forward	*C. butyricum*	GTAATGGAGGAAGCCACTTCGGT	Laurinavichene et al. 2010
Reverse	*K.pneumoniae*	TGCCCAGACCGATAACTTTA	Sun et al. 2010
Forward	*K.pneumoniae*	CTGTTTCTTCGCTTCACGG	Sun et al. 2010
Reverse	*L. rhamnosus*	GGAAGAACACCAGTGGCGAAGG	Larsen et al. 2013
Forward	*L. rhamnosus*	CAGGCGGAATGCTTAATGCGTTAG	Larsen et al. 2013

### Adsorption test

To investigate the role of extracellular polymeric substances (EPS) in the process of adsorption of cells to polyester nonwoven, a batch adsorption experiment was carried out. A cell suspension of 60 mL was taken from the chemostat, of which 30 mL was directly used for the adsorption test, and the remaining 30 mL was subjected to the removal of the EPS.

To get rid of EPS, cation exchange resin was employed (Frølund et al. 1996). In a nutshell, the cell suspension underwent three 0.1 M NaCl solution washes after being centrifuged at 4000*g* for 10 min. Reconstituted to its original volume, the washed cell pellet was then transferred to a 100-mL Erlenmeyer flask and mixed with 732 cation exchange resin (Beijing Solarbio Science & Technology Co., Ltd., China) at a ratio of 70 g/g of dry bacterial weight. For 3 h at 4 ℃, the Erlenmeyer flask was incubated on a shaker (200 rpm). The bacterial suspension was then transferred to a 50-mL centrifuge tube and centrifuged for 20 min at 12,000 rpm at 4 ℃. The supernatant was regarded as the EPS extracted from the cell suspension. The remaining cell pellet was resuspended to the original volume with sterile water and then used for the adsorption test.

According to the BCA protein assay Kit technique (Beijing Solarbio Science & Technology Co., Ltd., China) and the anthrone method with glucose as the standard, the exoprotein (PN) and exoploysaccharide (PS) concentrations in the EPS were examined on a UV/VIS spectrophotometer (Shimadze UV–VIS-NIR Spectrophotometer UV-3600) (Langer et al. 2018). In brief, under alkaline conditions, the protein will reduce Cu^2+^ to Cu^+^. Cu^+^ can form a blue-purple complex with BCA reagent, and its absorption value at 562 nm is determined. Compared with the standard curve, the concentration of the protein can be calculated. The standard curve was drawn as follows: according to the number of standard protein solutions (BSA protein standard reagent, 5 mg/mL), BCA working liquid (color developer) was prepared by adding 50 volumes of BCA reagent to 1 volume of Cu reagent (50:1) and thoroughly mixing. After that, a series of standard solutions at different concentrations (0, 10, 20, 30, 40, 50, and 60 mg/L) were prepared. BCA color developer of 5 mL was added into each standard solution of 0.2 mL, and the color was developed at 37 ℃ for 15–30 min. The absorbance was measured at 562 nm with a slit 3-mm quartz cuvette, and then the standard curve was obtained according to the absorbance vs. protein concentration. The sample instead of standard protein was used to determine its protein concentration according to the standard curve. The detailed information about the exoploysaccharide determination were as follows: An accurately weighed amount of anthrone (0.1 g) was added to a 100 mL of 80% sulfuric acid to dissolve it. A weighed amount of dried glucose (200 mg) was added to a 100 mL volumetric bottle with a concentration of 2000 mg/L. Then, a 10 mL solution was measured into a 100-mL volumetric bottle and prepared into a 200 mg/L glucose standard solution. After that, a series of standard solutions at different concentrations (0, 20, 40, 60, 80, 100, and 120 mg/L) were prepared, and each solution of 2 mL was put into 7-plug colorimetric tubes. The anthranone reagent of 6 mL was immediately added into each tube and mixed by shock. Each tube was heated in a boiling water bath for 15 min and quickly soaked in an ice bath to cool for 15 min. The absorbance values were quickly determined at 625 nm with a slit 10-mm quartz cuvette, and then a standard curve was obtained according to absorbance vs. glucose concentration. A sample of 2 mL was added into a dry and clean glass test tube, and the anthrone reagent of 6 mL was immediately added into the test tube. After heating and cooling like the above operation, the absorbance was measured at 625 nm with distilled water as the blank. The exoploysaccharide content was calculated according to the standard curve.

The cell suspension with EPS and the cell suspension without EPS were placed in a 100-mL serum bottle, respectively. Then several polyester nonwoven samples were added to the serum bottle and cultured for 4 h at 37 ℃. After that, the polyester nonwoven samples were taken out of the serum bottle, and a part of the samples was observed by scanning electron microscope (SEM) for bacterial adsorption. The other part of the samples was washed with 2 mL sterile water, and serial dilutions and plate counts were prepared to examine the change in bacterial adsorption amount before and after EPS removal.

### Scanning electronic microscope (SEM) analysis of cell adsorption

SEM was used to determine the morphological structure of the cells adhering to the polyester nonwoven. The polyester nonwoven samples were fixed using chemical methods as previously described (Wang et al. 2017). The samples were fixed with 2.5% glutaraldehyde overnight at 4 ℃ after being rinsed with 10 mM phosphate buffer. The samples were then dehydrated by passing them through repeated stages of 50%, 70%, 80%, 95%, and 100% ethanol before being dried in a vacuum dryer. For SEM observations, the dry sample was coated with gold powder and adhered to supports with silver adhesive. Then the samples were observed by scanning electron microscope (Quanta 450, FEI, USA).

## Results

### Establishment of a polyester nonwoven chemostat

**Fig. 2:**
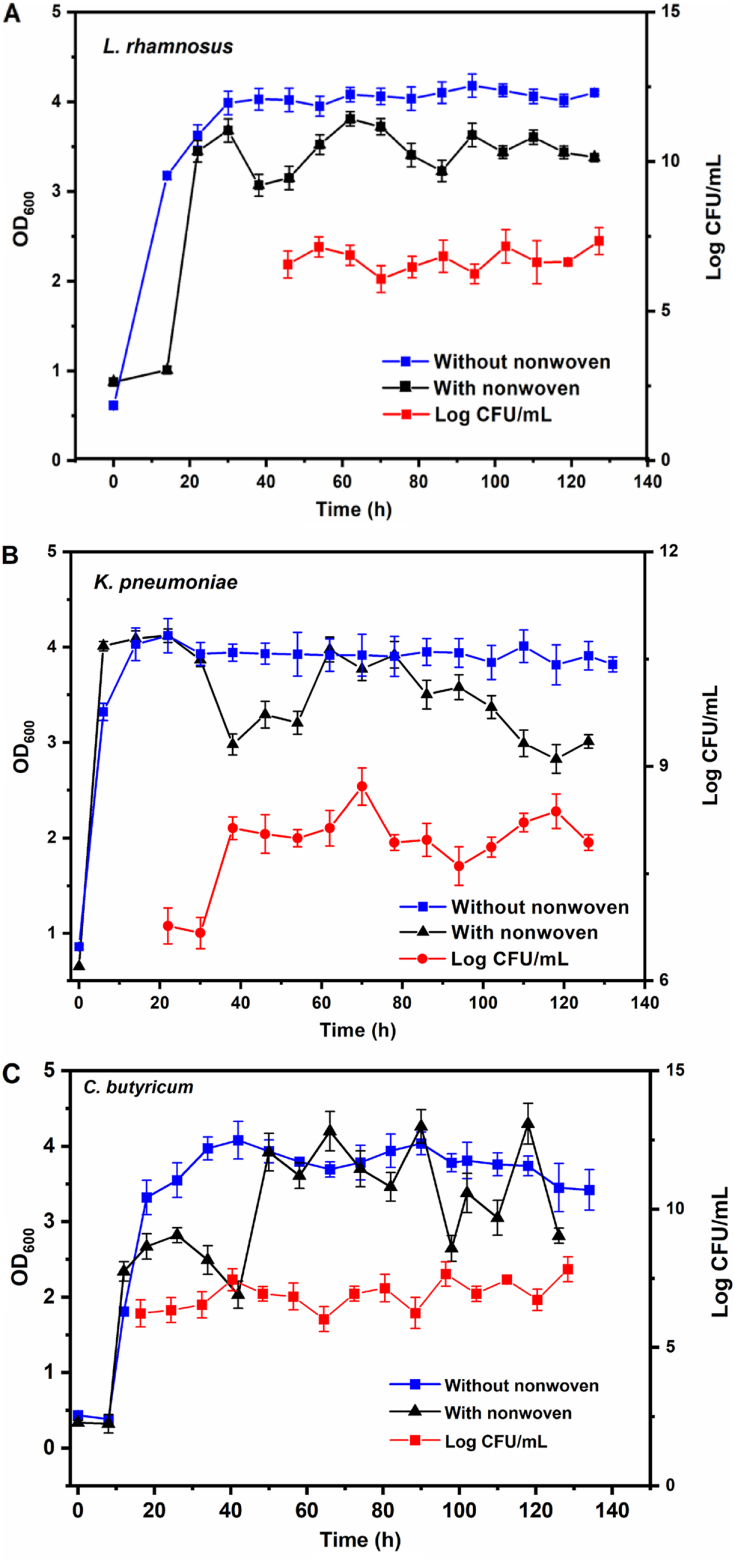
The dynamic relationship between OD (free cells) and the cell numbers (log CFU/mL, immobilized cells on the polyester nonwoven) of *L. rhamnosus* (**A**), *K. pneumoniae* (**B**) and *C. butyricum* (**C**) during the continuous fermentation

In this study, a polyester nonwoven was incorporated into a chemostat to immobilize the microbial cells. Switching from the chemostat (without polyester nonwoven) into the polyester nonwoven chemostat, the fluctuation of optical density (OD) for free cells was observed (Fig. 2).

In the continuous fermentation process of *L. rhamnosus*, the OD of free cells in the chemostat with or without polyester nonwoven kept increasing steadily during 0–30 h; however, the OD fluctuation of free cells in the polyester nonwoven chemostat was observed after 30 h (Fig. 2A). The maximum and minimum OD of *L. rhamnosus* reached 3.81 and 3.07 with the addition of polyester nonwoven into the chemostat, respectively. On the contrary, when the chemostat was run without polyester nonwoven, the OD of *L. rhamnosus* was stable at 3.96 ± 0.06 during the continuous culture. Comparing the standard deviations of the two groups of tests, it could be found that the standard deviation (SD) of the polyester nonwoven addition (0.15) was greater than that of non-addition (0.06), indicating that the addition of polyester nonwoven would cause distinct fluctuations of free cells. The same result was also presented when *K. pneumonia* and *C. butyricum* were cultured in polyester nonwoven chemostat. For *K. pneumoniae* and *C. butyricum*, after a period of steady growth, the OD of free cells began to show difference. The OD of *K. pneumoniae* was stable at 3.92 ± 0.05 in chemostat without polyester nonwoven (SD = 0.05), but after adding the polyester nonwoven, the OD of *K. pneumoniae* varied from 2.83 to 3.92 (SD = 0.39) (Fig. 2B). The OD of *C. butyricum* was stable at 3.82 ± 0.17 in chemostat without nonwoven (SD = 0.17) and had a fluctuation, varying from 2.03 to 4.30 after adding the nonwoven in the chemostat (SD = 0.73) (Fig. 2C). Simultaneously, when the cell numbers on the polyester nonwoven increased, the OD value of free cells decreased (Fig. 2). The result showed that the fluctuations of free cells might be related to the adsorption and desorption of cells on the polyester nonwoven.

### Adsorption of cells on the polyester nonwoven

**Fig. 3:**
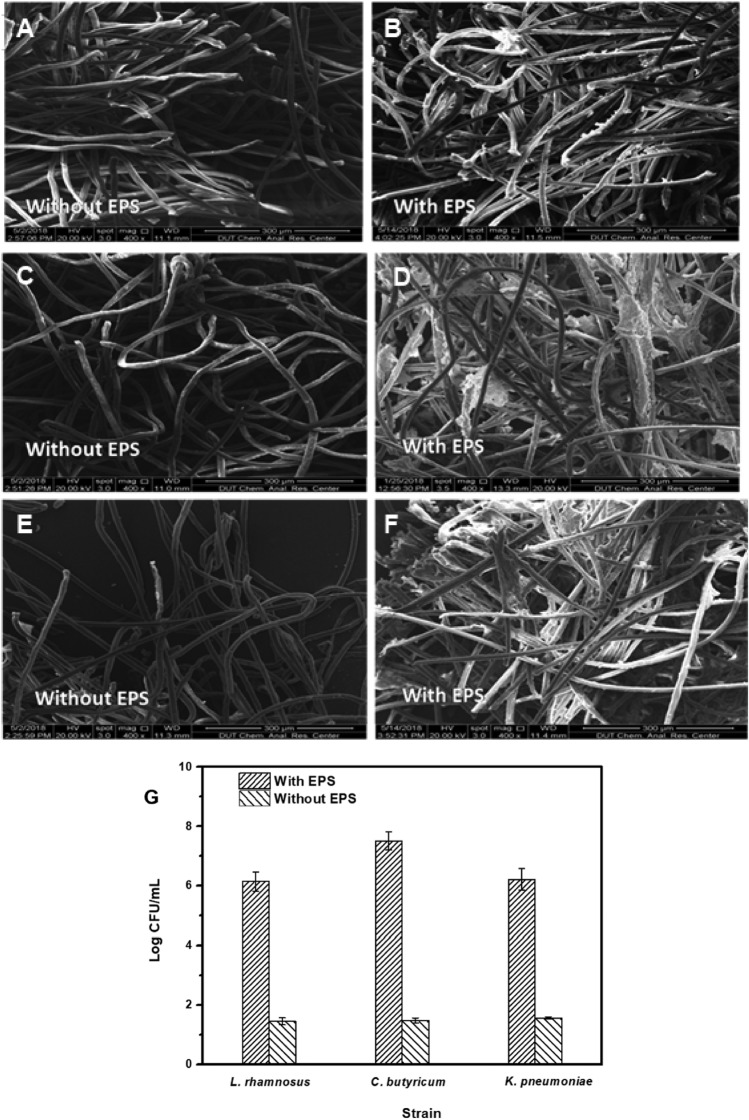
Scanning electron microscope of *L. rhamnosus* (**A**, **B**), *K. pneumoniae* (**C**, **D**) and *C. butyricum* (**E**, **F**) on polyester nonwoven with EPS or without EPS, as well as the comparison of the cell numbers on the polyester nonwoven between with EPS and without EPS (**G**)

**Fig. 4:**
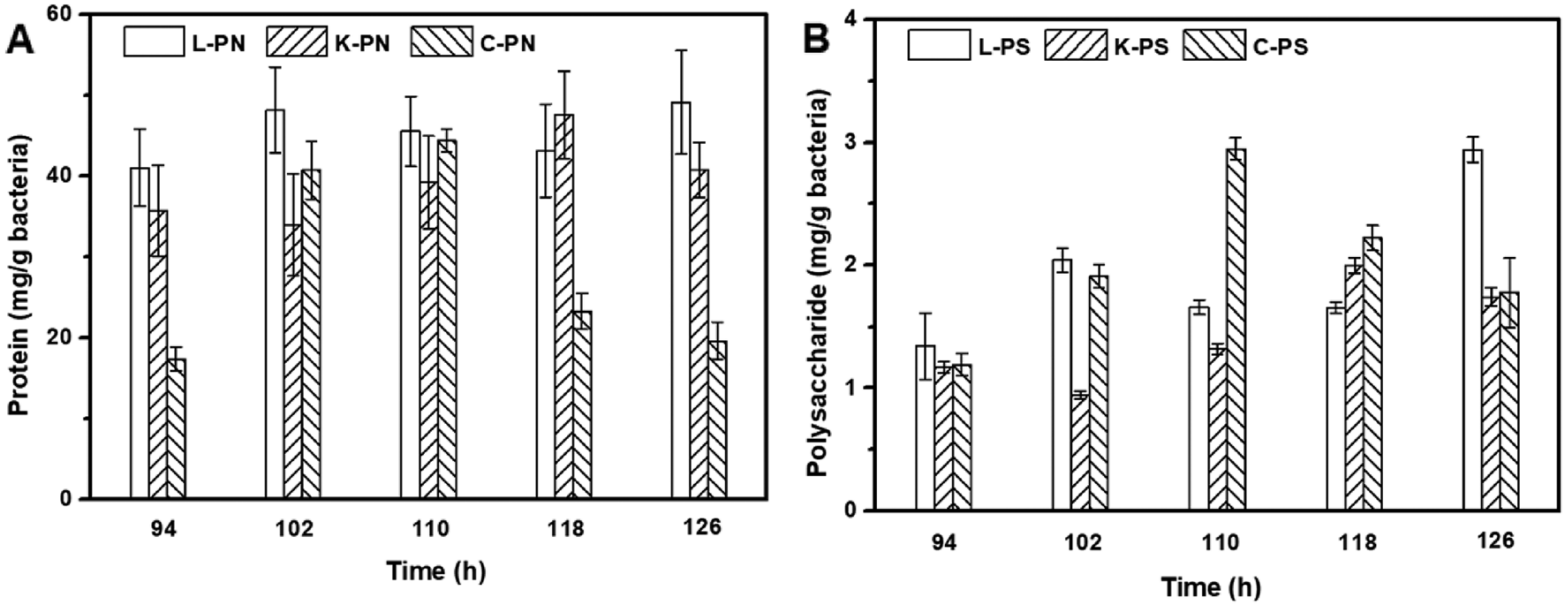
The exoprotein (**A**) and exopolysaccharide (**B**) content of biofilm on polyester nonwoven (PN: exoprotein, PS: exopolysaccharide, L: *L. rhamnosus*, K: *K. pneumoniae*, C: *C. butyricum*)

To analyze the adsorption of cells on the polyester nonwoven, an adsorption test was performed. According to SEM, only a small number of bacterial cells could be observed on the polyester nonwoven filaments after removing the EPS (Fig. 3A, C, E), whereas many bacterial cells encapsulated with EPS could be observed on the polyester nonwoven filaments (Fig. 3B, D, F). By washing with 2 mL sterile water and preparing serial dilutions and plate counts to measure the cell number after the adsorption experiment, the cell numbers on polyester nonwoven of *L. rhamnosus*, *K. pneumoniae*, and *C. butyricum* with EPS were 6.14, 7.51, and 6.21 log CFU/mL, respectively. After getting rid of EPS, the cell numbers of *L. rhamnosus*, *K. pneumoniae*, and *C. butyricum* were 1.45, 1.48, and 1.56 log CFU/mL, respectively (Fig. 3G). According to above results, the cell numbers on the polyester nonwoven with EPS were higher than those removing EPS. Thus, EPS were important for the attachment of cells. In addition, the exoprotein (PN) and exoploysaccharide (PS) as the major composition of EPS were positively correlated to the biofilm formation (Wang et al. 2006). Therefore, the content of PN and PS was measured in this study. During the continuous fermentation process, the protein content of the three strains is significantly higher than that of the polysaccharide (Fig. 4), which is consistent with the results of a previous study (Zhang et al. 2007).

### Desorption of cells on the polyester nonwoven

**Fig. 5:**
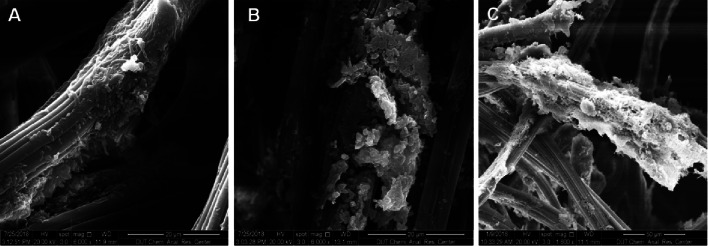
The formation of multilayer biofilm by *L. rhamnosus* (**A**), *K. pneumoniae* (**B**) and *C. butyricum* (**C**) on the polyester nonwoven

**Fig. 6:**
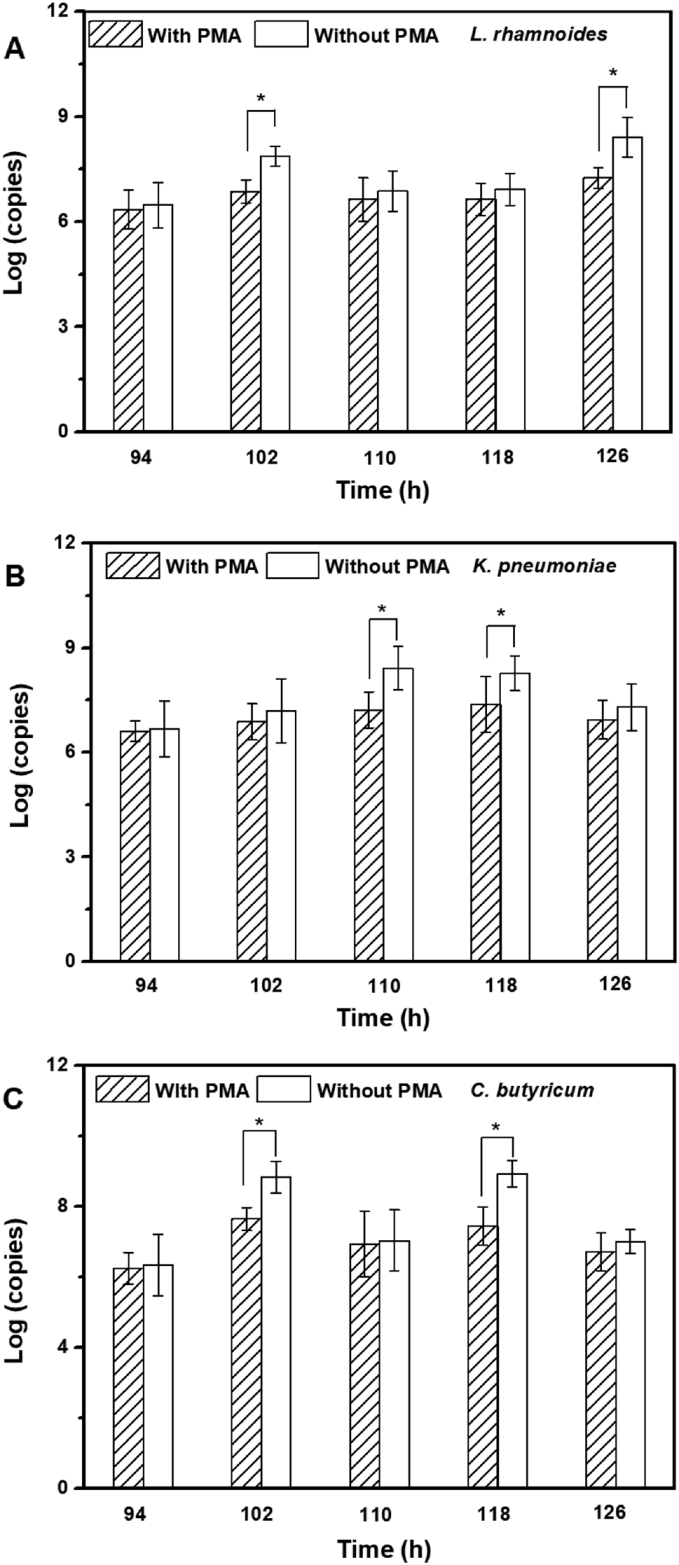
Change in copy number of bacteria (A: *L. rhamnosus*, B: *K. pneumoniae*, C: *C. butyricum*) on the polyester nonwoven with PMA treatment and without PMA treatment. Error bars represent the standard deviation around the mean. Variation in the content was determined to be significant (*p* < 0.05) using Student’s *t*-test

According to the scanning electronic microscope of biofilm on the polyester nonwoven (Fig. 5), bioaggregates could be clearly observed on the polyester nonwoven after the formation of multilayer biofilms. Simultaneously, the phenomenon of bacterial death, which could cause the desorption of bacterial cells, was observed using the method of PMA combined with qPCR (Fig. 6). The log number of copies for *L. rhamnosus* significantly decreased from 7.87 to 6.96 (*p* = 0.04 < 0.05) at 102 h after PMA treatment, and at 126 h, the log number of copies for *L. rhamnosus* significantly decreased from 8.41 to 7.39 (*p* = 0.03 < 0.05). Furthermore, the proportion of death cells was 87.7% and 90.5% at 102 h and 126 h, respectively. After PMA treatment, the log number of copies for *K. pneumoniae* significantly changed from 8.42 to 7.21 (*p* = 0.03 < 0.05) and 8.27 to 7.37 (*p* = 0.04 < 0.05) at 110 h and 118 h, respectively. The proportion of death cells in *K. pneumoniae* was 93.8% and 87.4% at 110 h and 118 h, respectively. Moreover, the log number of copies for *C. butyricum* also declined from 8.44 to 7.64 (*p* = 0.04 < 0.05) and 8.93 to 7.44 (*p* = 0.02 < 0.05) at 102 h and 118 h, respectively, indicating that the proportion of death cells was 83.7% and 91.8% at 102 h and 118 h, respectively. Thus, the results demonstrated that there were a lot of dead cells on the polyester nonwoven, and these dead cells would detach from the polyester nonwoven.

## Discussion

For industrial applications, the immobilization of industrial microbial cells is a prospective technique to enhance the productivity and stability of fermentation. Among the cell immobilization methods, the adsorption is obviously easier due to no need for specialized equipment and reasonable price. Furthermore, the adsorption can enable direct interaction between nutrients and immobilized cells, leading to a high mass transfer and substrate conversion efficiency (Giese et al. 2020; Sekoai et al. 2018). The selection of a simple and efficient carriers for microbial immobilization is essential for the performance of the chemostat. In this study, the polyester nonwoven was suitable for cell immobilization. After about 20 h, the cell numbers on polyester nonwoven can reach 6.5 ± 0.38 log CFU/mL (Fig. 2). The cell adsorption time on polyester nonwoven is shorter than that on cellulose foam and disk carriers, i.e. 72 h (Fujii et al. 2001; Żur et al. 2020). In addition, polyester nonwovens do not need to be pretreated before cell adsorption, in contrast to biochar and polyurethane foam (Lou et al. 2019; Kurade et al. 2019). Thus, more and more research was focused on the use of the polyester nonwoven to immobilize various cells and enzymes (Shim et al. 2017; Lee et al. 2019; Zhang et al. 2021b). The polyester nonwoven was shown to be advantageous in cell immobilization.

In the polyester nonwoven chemostat, the fluctuation of OD for free cells was observed, and it was related to the adsorption and desorption of bacterial cells on the polyester nonwoven. In general, hydrogen bonds, ionic and hydrophobic interactions, and van der Waals forces are the principal interactions between the adhered cells and material surfaces (Rodríguez‑Restrepo et al. 2020). In the case of polyester nonwovens, the cells might be adsorbed on the nonwoven surface by the van der Waals force and the hydrophobic interaction at the early stage of adsorption because of the hydrophobicity of nonwoven and cell surfaces. Furthermore, it is possible to match the adsorption parameters for cells on polyester nonwoven to the Freundlich and Langmuir isotherms according to the previous studies (Mortazavi and Aghaei 2020; Aghaei et al. 2020). After the initial stage of adsorption, the EPS secreted by cells was important for the further adsorption of bacterial cells on the polyester nonwoven (Fig. 3). Bacterial cells are likely to show a dynamic double-layered EPS structure as this biogenic glue immobilizes single cells in multicellular aggregates (Vlaeminck et al. 2010), and EPS formation is necessary for the adsorption of cells onto the carriers to form biofilm (Wang et al. 2017). In addition, the content of exoprotein in the EPS was higher (Fig. 4). Exoprotein with negatively charged amino acids are involved in electrostatic bonds with multivalent cation, thus decreasing the negative surface charge density surrounding the cell surface and assisting in cell aggregation (Zhang et al. 2007). Overall, exoprotein was not only an important factor in forming stable aggregate structure but also beneficial for bacterial adhesion. A previous study showed that free cells could adhere to the interface of polyester nonwoven because they secreted EPS, and detachment of biofilm would occur when biofilm matured (Zhu et al. 2015). The desorption of bacterial cells was explained to help us further understand the fluctuation in the process of chemostat culture. Some factors have been suggested to be important in biofilm detachment, including nutrient levels, microbial growth status, quorum-sensing signals, c-di-GMP concentration, and the activation of a lytic bacteriophage (Hunt et al. 2004). Many reports exhibited that content of c-di-GMP had a significant influence on secreting exoprotein and exopolysaccharide (Zhang et al 2021a; Cao et al. 2022). In this study, the major factor for the detachment of biofilms was that the formation of multilayer biofilms might limit the transport of substrate, which would lead to the death of cells on the polyester nonwoven (Figs. 5, 6). The transport of substrate plays a crucial role in biofilm development since the content of nutrients determines the growth of cells (Picioreanu et al. 2000). Overall, the balance between adsorption and desorption of bacterial cells on the polyester nonwoven was the key factor leading to the fluctuation of free cells. The phenomenon of viable cell adsorption and dead cell desorption on the carries can reflect the high efficiency of the polyester nonwoven.

## Conclusions

The cell immobilization on polyester nonwoven was a rapid and cheap adsorption process without pretreatment. The adsorption and desorption qualities of cells were linked to the fluctuation of bacterial cells when polyester nonwoven was added to the chemostat for cell immobilization. As a large portion of EPS, exoprotein is crucial for attachment, whereas the biofilm detachment was linked to the development of multilayer biofilm and the death of cells on the polyester nonwoven. The construction of a chemostat made of polyester nonwoven revealed critical information on the dynamic balance of cell adsorption and desorption on polyester nonwoven.

## Data Availability

Data may be made available on request.
